# A stepped wedge cluster randomised trial of a cognitive remediation intervention in alcohol and other drug (AOD) residential treatment services

**DOI:** 10.1186/s12888-019-2044-4

**Published:** 2019-02-13

**Authors:** Jamie Berry, Isabella Jacomb, Jo Lunn, Antoinette Sedwell, Anthony Shakeshaft, Peter J Kelly, Pooria Sarrami, Megan James, Skye Russell, Talia Nardo, Daniel Barker, Jennifer Holmes

**Affiliations:** 1 Macquarie University, Balaclava Road, North Ryde, NSW 2109 Australia; 2 Advanced Neuropsychological Treatment Services, PO Box 4070, Strathfield South, NSW 2136 Australia; 3 We Help Ourselves, PO Box 1779, Rozelle, NSW 2039 Australia; 4 Agency for Clinical Innovation, Level 4, 67 Albert Ave, Chatswood, NSW 2067 Australia; 5 National Drug and Alcohol Research Centre, University of New South Wales, Sydney, NSW 2052 Australia; 6 Illawarra Health and Medical Research Institute and the School of Psychology, Faculty of Social Sciences, University of Wollongong, Building 41, Room 128, Wollongong, NSW 2522 Australia; 7 South Western Sydney Clinical School, University of New South Wales, Goulburn St, Liverpool, NSW 2170 Australia; 8 University of Newcastle, University Drive, Callaghan, NSW 2308 Australia; 9 Ministry of Health NSW, 73 Miller St, North Sydney, NSW 2060 Australia

**Keywords:** Cognitive rehabilitation, Cognitive remediation, Alcohol and drug, Executive function, Treatment retention, Goal setting

## Abstract

**Background:**

Executive functioning impairment is common in substance use disorder and is a major risk factor for poor treatment outcomes, including treatment drop-out and relapse. Cognitive remediation interventions seek to improve executive functioning and offer a promising approach to increase the efficacy of alcohol and other drug (AOD) treatments and improve long-term therapeutic outcomes. This protocol describes a study funded by the NSW Agency for Clinical Innovation that assesses the effectiveness of delivering a six-week group-based intervention of cognitive remediation in an ecologically valid sample of people attending residential AOD treatment services. We primarily aim to investigate whether cognitive remediation will be effective in improving executive functioning and treatment retention rates. We will also evaluate if cognitive remediation may reduce long-term AOD use and rates of health service utilisation, as well as improve personal goal attainment, quality of life, and client satisfaction with treatment. In addition, the study will involve an economic analysis of the cost of delivering cognitive remediation.

**Methods/design:**

The study uses a stepped wedge cluster randomised design, where randomisation will occur at the cluster level. Participants will be recruited from ten residential AOD treatment services provided by the non-government sector. The intervention will be delivered in 12 one-hour group-based sessions over a period of six weeks. All participants who are expected to receive treatment for the duration of the six-week intervention will be asked to participate in the study. The clusters of participants who are randomly assigned to the treatment condition will complete cognitive remediation in addition to treatment as usual (TAU). Primary and secondary outcome assessments will be conducted at pre-cognitive remediation/TAU phase, post-cognitive remediation/TAU phase, two-month follow-up, four-month follow-up, six-month follow-up, and eight-month follow-up intervals.

**Discussion:**

This study will provide comprehensive data on the effect of delivering a cognitive remediation intervention within residential AOD treatment services. If shown to be effective, cognitive remediation may be incorporated as an adjunctive intervention in current treatment programs.

**Trial registration:**

Australian and New Zealand Clinical Trials Register (ANZCTR): ACTRN12618001190291. Prospectively registered 17th July 2018.

## Background

Substance use disorder (SUD) is a major global health problem, which has increased notably in the last few years [1]. In Australia, a recent publication reported that one in 200 people in the general population received treatment for substance use in 2014–15, which represents a 6% increase from the previous year [2]. Although SUD is frequently described as a chronic and relapsing condition, favourable long-term outcomes are consistently associated with alcohol and other drug (AOD) treatment completion [3]. However, failure to complete treatment, or “drop-out”, is very common in both inpatient and outpatient treatment services [4, 5].

According to a systematic review of the literature, one of the major risk factors of drop-out from AOD treatment is cognitive impairment [6]. Over the last two decades, neuropsychological research has consistently shown that substance use is associated with a significant degree of impairment in cognitive functioning. Whilst specific aspects of cognitive functioning may predate and influence the initiation of AOD use [7], increased substance use results in a greater degree of impairment [8, 9]. That is, there is a strong association between the quantity and duration of AOD use and the degree of cognitive dysfunction. Cognitive deficits in individuals with SUD may be further exacerbated by the presence of common comorbidities such as traumatic brain injury and psychiatric disorders [10, 11]. Although partial recovery of cognitive impairment may occur after immediate cessation of AOD use, many cognitive deficits fail to recover following withdrawal and abstinence [12, 13]. For example, it has been estimated that approximately 50–80% of recently detoxified individuals with SUD are cognitively impaired when assessed with the Montreal Cognitive Assessment [14–17].

In individuals with SUD, the most prominent cognitive deficits are seen in executive functioning (EF) [9, 18]. EF abilities (such as working memory, planning and organisational skills, reasoning, and self-regulation) are mediated by the frontal cortex, an area of the brain that is particularly vulnerable to substance-related impairment [19]. Common AOD treatment interventions, including counselling and psychoeducation, are heavily reliant on intact EF abilities in order to identify, promote and achieve successful behavioural change [20, 21]. Thus, EF impairment significantly predicts treatment drop-out and relapse [22–24].

Given the clinical relevance of EF deficits in the SUD population, it has been proposed that evidence-based neuropsychological interventions may be particularly useful in improving treatment outcomes and facilitating recovery [25–27]. Traditionally, cognitive remediation (CR) interventions have been inspired by two main theoretical perspectives; restoration and compensation [28]. Restorative (or “bottom-up”) approaches attempt to repair impaired cognitive skills directly by using “drill and practice” exercises, and have typically been used in mental health populations [29]. On the other hand, compensatory (or “top-down”) techniques circumvent the deficit with reliance on intact cognitive skills and environmental and prosthetic supports, and are frequently employed in acquired brain injury rehabilitation [30].

In the SUD literature, the majority of existing studies have predominantly used a restorative approach to CR, generally via “drill and practice” computerised tasks [31–35]. Whilst the results of these studies suggest that “drill and practice” leads to improvement on performance-based measures of EF, whether these improvements also lead to demonstrated improvement in everyday functioning is unclear. Performance-based measures of EF show limited ecological validity and do not relate to everyday functioning as well as inventory-based measures [36, 37]. Furthermore, there is intense debate as to the precise mechanisms of “drill and practice” training and the ability of this approach to transfer trained skills into real-world improvements, most notably treatment retention and recovery [25, 38].

In comparison, meta-analytical studies and systematic reviews have shown that compensatory CR approaches have significantly stronger effects not only on global cognition, but also on real-world functioning, than programs that focus only on restorative techniques in other cognitively impaired populations [30, 39, 40]. Whilst only a limited number of studies have examined the effects of compensatory CR in the SUD population, preliminary findings are promising [41–44]. In a sample of patients with severe alcohol-related brain damage, an intensive rehabilitation program incorporating compensatory CR strategies resulted in an 85% reduction in subsequent acute admissions [44]. Two other studies have assessed the efficacy of Goal Management Training (GMT), a predominantly “top-down” CR technique, combined with mindfulness meditation [41, 43]. Results revealed that GMT not only improved performance on tasks of working memory, impulsivity, and decision making, but also on an ecologically valid measure of goal-directed behaviour, suggesting that the intervention relates to improvements in daily life activities [43]. Another recent study showed that residents in community AOD treatment who received a combination of restorative and compensatory CR showed improvement on a performance-based measure of inhibition, as well as inventory-based measures of EF, impulsivity, self-control and quality of life [42]. Overall, these initial results support the argument that a “top-down” approach to CR may translate to real-world situations because people are better able to transfer skills from the training environment into their daily lives and that teaching such strategies helps patients compensate for the effects of persistent cognitive impairments on functioning [45, 46].

A major limitation in the existing literature is that there have been no randomised trials of compensatory CR in the SUD population. Another difficulty of the literature is that studies often exclude participants with common comorbidities such as traumatic brain injury and psychiatric comorbidities. This ultimately affects the generalisability of results. In addition, many studies have relied on performance-based measures of EF rather than more ecologically valid EF measures, which have been shown to be better predictors of real-world functioning and treatment success [47]. Finally, previous SUD literature has not followed-up the long-term effects of compensatory CR, such as treatment retention and recovery. Given findings of its success in improving functional outcomes in other cognitively impaired groups, we propose that compensatory CR aimed at improving EF is likely to be a beneficial adjunctive intervention to boost AOD treatment efficacy and long-term outcomes.

### The current study

The study will aim to examine the effectiveness of adding a CR intervention to TAU on two primary outcomes, being:

1. A self-report inventory of executive functioning; and
2. Treatment retention

The study will also assess the impact of CR in addition to TAU on a number of secondary outcomes:

1. AOD use
2. Health service utilisation
3. Functional goal attainment
4. Quality of life
5. Client satisfaction with treatment
6. Performance-based measures of EF
7. Economic analysis

The study will be conducted as a stepped wedge randomised cluster trial. Stepped wedge designs are increasingly being utilised in the evaluation of interventions within routine treatment services and are recommended where there are limited numbers of clusters [48–50]. The study is funded by the NSW Agency for Clinical Innovation.

## Methods/design

### Study setting

The research program will be conducted at ten residential treatment facilities run by non-governmental organisations in NSW, Australia. All organisations will be abstinence-based however a portion of residents may be prescribed medications or opioid substitution treatments (OST) (e.g. methadone). Limited leave from the residential facilities, close observation by staff and other residents, and random urine tests will ensure abstinence from substances while participants are enrolled in treatment programs. Services that accommodate at least 15 residents and have a minimum project duration of 10 weeks will be invited to participate in the study.

### Ethics approval and consent to participate

The Macquarie University Human Research Ethics Committee (HREC (Medical Science)) approved the research to trial (Reference Number 5201800077), which is registered with the Australian New Zealand Clinical Trials Registry (ACTRN12618001190291). All participants will provide written informed consent to a research officer prior to participating in the trial. Consent will be provided separately for access to linked healthcare data through the Centre for Health Record Linkage. Participants will also able to request to withdraw from the study at any point during their participation (data collection, intervention or both).

### Study design

The proposed study will be conducted as a prospective stepped wedge cluster randomised trial, where randomisation will occur at the cluster level. The design involves random and sequential crossover of clusters from TAU to CR until all clusters have implemented CR (Table 1). TAU was selected as the comparator condition as the CR intervention must be demonstrated to be effective above and beyond what residential AOD services are currently offering in order to be useful as an adjunct treatment. There will be four six-week phases of either TAU or CR, interspersed by two-week test phases, at each of the ten sites. The order in which the sites receive cognitive remediation will be generated by an independent statistician using computer-generated randomisation. After the initial two-week assessment phase (weeks 1–2), all clusters will undergo 6 weeks of treatment as usual (weeks 3–8). Following the second two-week assessment phase (weeks 9–10) clients at the three sites randomised to start first will receive CR for a period of 6 weeks (weeks 11–16), then this treatment will continue for the life of the study. After the next two-week test phase (weeks 17–18), another 3 sites will be randomly selected to commence CR, and so on until all services have provided CR. Six weeks after the final post-intervention phase (weeks 33–34) an additional two-week testing phase will be allocated for follow-up (weeks 41–42). CONSORT procedures will be followed including using an intention to treat analysis [51].

**Table 1:** Progression of sites from Treatment as Usual to Cognitive Remediation In Stepped Wedge Design

	Test Phase 1	Period 1	Test Phase 2	Period 2	Test Phase 3	Period 3	Test Phase 4	Period 4	Test Phase 5	Period 5	Test Phase 6
Week	1–2	3–8	9–10	11–16	17–18	19–24	25–26	27–32	33–34	35–40	41–42
Site 1	Test Phase 1	TAU 1	Test Phase 2	Cog Rem 1	Test Phase 3	Cog Rem 2	Test Phase 4	Cog Rem 3	Test Phase 5	F/U Period	Test Phase 6
Site 2		TAU 1		Cog Rem 1		Cog Rem 2		Cog Rem 3		F/U Period	
Site 3		TAU 1		Cog Rem 1		Cog Rem 2		Cog Rem 3		F/U Period	
Site 4	Test Phase 1	TAU 1	Test Phase 2	TAU 2	Test Phase 3	Cog Rem 1	Test Phase 4	Cog Rem 2	Test Phase 5	F/U Period	Test Phase 6
Site 5		TAU 1		TAU 2		Cog Rem 1		Cog Rem 2		F/U Period	
Site 6		TAU 1		TAU 2		Cog Rem 1		Cog Rem 2		F/U Period	
Site 7	Test Phase 1	TAU 1	Test Phase 2	TAU 2	Test Phase 3	TAU 3	Test Phase 4	Cog Rem 1	Test Phase 5	F/U Period	Test Phase 6
Site 8		TAU 1		TAU 2		TAU 3		Cog Rem 1		F/U Period	
Site 9		TAU 1		TAU 2		TAU 3		Cog Rem 1		F/U Period	
Site 10		TAU 1		TAU 2		TAU 3		Cog Rem 1		F/U Period	

Note: *Cog Rem* Cognitive Remediation Phase, *TAU* Treatment-As-Usual Phase, *F/U Period* Follow Up Period

The estimated number of participants is based on prior research in NSW residential drug and Alcohol Treatment [42]. *Discharge* refers to the discharge of participants from the residential treatment service (either through completing the program or self-discharge/drop out before the completion of the program). *Attrition* refers to drop-out from the study or otherwise being non-contactable following discharge from the treatment service.

1. Each site will start with *n* = 20 participants on average;
2. Within a single cohort, there will be:
  - a 40% discharge rate over each six-week TAU/CR phase plus two-week test phase;
  - a 65% discharge rate over the following 2 months;
  - an 80% discharge rate over the following 4 months;
  - a 90% discharge rate over the following 6 months; and
  - a 95% discharge rate over the following 8 months;
3. New clients will enter a service at the same rate as other clients leave the service;
4. If a client enters a service during a TAU/CR phase, they will delay their participation in the trial until the next test phase;
5. Some clients will participate in more than one CR phase;
6. All participants who have completed at least one TAU/CR phase will be eligible for follow-up testing, which will occur at test phases 3, 4, 5 and 6.
7. If a participant has had a CR phase they are ineligible for TAU follow-up.
8. If a participant has had more than one CR phase they will still be eligible for CR follow-up
9. Follow-up attrition rates have been estimated to be:
  - 65% attrition at two-month follow up
  - 75% attrition at four-month follow up
  - 85% attrition at six-month follow up
  - 90% attrition at eight-month follow up

### Participants

All participants will be attending residential AOD treatment programs in NSW. It is expected that over the course of the study approximately 440 participants will be recruited, with 40% expected to drop out within the first 8 weeks. Based on figures from 2014 to 15, we anticipate that the majority of participants will be seeking treatment for the four most common principal drugs of concern; alcohol, cannabis, amphetamines and heroin [2]. All participants will provide informed written consent and will be aged 18 years or older.

### Inclusion and exclusion criteria

All participants attending the residential programs will be invited to participate in the study. Exclusion criteria will be kept to a minimum to ensure that the study can examine the effectiveness of using CR in addition to TAU within a “real world” setting. For example, traumatic brain injury and psychiatric comorbidities are common in individuals with AOD use [10, 11], therefore these factors will not be part of the exclusion criteria in order to make the sample as representative as possible. Similarly, while the residential treatment services will be abstinence-based, a portion of clients will be prescribed OST or other medication, and these participants will also be included.

### CR intervention

The manualised Cognitive Remediation in Drug and Alcohol Services (CRiDAS) program will be used for CR, which consists of 12 one-hour group-based sessions that will take place across 6 weeks (two sessions per week). Each session will be dedicated to strategy training, which includes traditional instructional pedagogical approaches, group discussion/reflection and exercises to demonstrate concepts. The CR intervention was developed with a strong emphasis on the training of EF in view of the finding that EF is particularly impaired in an AOD treatment population. Barkley’s evolutionary model of EF [52, 53] was used to structure a large part of the program. The CR program will use a “top-down” approach, including training in compensatory strategy use with a particular emphasis on EF. The modules cover the following topics: brain functioning, attention, memory, visual and verbal working memory, emotion regulation, decision-making and problem-solving. Facilitators will be staff of the residential AOD treatment sites who will undergo training to administer the intervention, and will be chosen by service management on the basis of a minimum skillset relevant to program delivery. Facilitators will be trained immediately prior to introducing the intervention, to prevent staff from utilising CR techniques during phases of TAU.

Intervention facilitators will take attendance for each module of the intervention in order to monitor adherence and dose of the intervention. An independent researcher/clinician will use the CRiDAS treatment fidelity measure (to be developed) to measure fidelity via audio/video recordings of 10% of the sessions at each site.

### Assessment measures

See Table 2 for an outline of the timeline of assessment measures. To provide an estimate of the overall level of premorbid intellectual functioning, the Test of Premorbid Functioning [54] will be administered once only, at the first test phase. One primary outcome measure will be an inventory-based measure of EF, as previous findings have shown that inventory-based assessment is more strongly associated with real-world functioning outcomes in SUD than performance-based assessment [36]. Given the high rates of treatment drop-out in residential AOD treatment services, another primary outcome measure will be the length of treatment retention, measured as time in treatment as a proportion of program duration [4]. This is because the program duration may vary across services. Secondary outcome measures will evaluate substance use severity, health service utilisation, individual goal-attainment, quality of life, treatment satisfaction, and performance-based measures of EF. An economic analysis will also be undertaken to evaluate the costs associated with delivering CR versus TAU. Data collection will be facilitated by research assistants blinded to the randomised condition, that is, whether or not a site has implemented cognitive remediation.

**Table 2:** Timeline of Assessment Measures

Domain assessment and instrument used	Baseline Assessment	During Intervention	Post-TAU/CR	Follow-Up	Post-trial
Mini International Neuropsychiatric Interview or DSM-V	X				
Drug and Alcohol Cognitive Impairment Screening Tool (DACIST)	X				
Australian Treatment Outcomes Profile (ATOP)	X			X^a^	
Severity of Dependence Scale (SDS)	X		X	X	
Test of Premorbid Functioning	X				
Behaviour Rating Inventory of Executive Functioning – Adult Version (BRIEF-A)	X		X	X	
Kessler Psychological Distress Scale Plus (K10+)	X		X	X	
Quality of Life Enjoyment and Satisfaction Questionnaire – Short Form (Q-LES-Q-SF)	X		X	X	
Patient-Reported Outcomes Measurement Information System Global 10 (PROMIS Global-10)	X		X	X	
Brief Self-Control Scale	X		X	X	
EUROQOL – EQ-5D (EQ-5D)	X		X	X	
EUROHIS-QOL 8	X		X	X	
Q-LES-Q-SF	X		X	X	
Alpha Span Task	X		X	X	
Symbol Digit Modalities Test (SDMT)	X		X	X	
Goal Attainment Scale 2.0 (GAS 2.0)	X		X	X	
Brief Executive Assessment Tool (BEAT)	X		X	X	
Stroop Task	X		X	X	
Five-Point Test	X		X	X	
Group Session Rating Scale		X			
Feasibility/satisfaction questionnaire (staff and clients)					X
Time in Treatment (for individuals)					X
Mean Length of Stay as a proportion of program duration for sites	X				X
Health Service Utilisation					X
Economic Analysis					X

Notes: ^a^ = Post-discharge follow-up only

Outcome measures will be taken at intake, the test phases (2 week periods pre- and post- TAU/CR phases), and follow-up (2 months, 4 months, 6 months and 8 months following completion of a TAU/CR phase). Attempts to improve follow-up rates in the current study will include using telephone interviews and online questionnaires at follow-up to administer some of the assessments if necessary.

### Primary outcome measures

#### Behavior Rating Inventory of Executive Function – Adult Version (BRIEF-A) [55]

The BRIEF-A is a 75 item self-report questionnaire consisting of nine subscales. The Global Executive Composite (GEC) provides an overall summary score and will be a primary outcome variable.

#### Time in treatment

Duration of treatment will be provided by the Alcohol and Other Drug Treatment Services National Minimum Data Set [56]. Time spent in treatment (length of stay) as a proportion of program duration will be used as a primary outcome variable.

### Secondary outcome measures

#### Severity of Dependence Scale (SDS) [57]

The SDS is a 5-item questionnaire which assesses for severity of substance use. The outcome variable will be total score.

#### Health service utilisation

For each participant, the number of visits to health services in the year prior to and following commencement of CR will be compared. These data will be obtained from linking data obtained from this study with four other datasets (NSW Admitted patient data collection, NSW Emergency Department data collection, NSW Cause of death unit record file, NSW mental health ambulatory data collection). Data linkage will be undertaken by the Centre for Health Record Linkage. Variables of interest will include medical centre, hospital and emergency room admissions, access of community-based mental health and AOD services, and mortality rates.

#### Brief Executive Assessment Tool **(**BEAT) [58]

The BEAT is a screening tool developed to be sensitive to executive dysfunction, particularly in a SUD population. It includes both performance- and inventory-based items. The outcome variable will be total score.

#### Stroop task [59]

In this measure of response inhibition, participants must respond as quickly and accurately as possible across three conditions. The first condition presents the words “red”, “blue”, and “green”; the second presents patches of colours; the third presents words printed in incongruent colours and requires the participant to ignore the word and say the colour. The outcome variable will be total score on the third condition.

#### Five-point test [60]

The Five-Point Test assesses non-verbal figural fluency and consists of producing novel designs under time constraints. The task consists of a sheet of paper with 40 five-dot matrices. Participants are asked to produce as many different figures as possible by connecting the dots within each rectangle within 3 min. The outcome variable will be total designs correctly completed.

#### Alpha span task [61]

The Alpha Span Task is a measure of working memory in which participants are read a list of words and are asked to say the words back in alphabetical order. The outcome variable will be the total alpha score.

#### Symbol Digit Modalities Test (SDMT) [62]

The SDMT is a measure of processing speed in which the participant writes a series of numbers corresponding to symbols according to a symbol-number key at the top of the page. The participant is asked to work through a series of symbols as quickly as they can, with the outcome variable the total number of symbols that can be translated in 90 s.

#### Brief Self-Control Scale (BSCS) [63]

The BSCS is a 13-item self-report questionnaire that assesses individual differences in the construct of self-control. The outcome variable will be total score.

#### EUROQOL – EQ-5D-5L (EQ-5D) [64]

The EQ-5D is a 5-item measure of the quality of life that can be used to calculate Quality Adjusted Life Years in people with Substance Use Disorders. The outcome measure will be total score.

#### EUROHIS-QOL 8-item index (EUROHIS-QOL 8) [65]

The EUROHIS-QOL 8 is an 8-item measure of the quality of life across four domains (physical, psychological, environment, and social). Total QOL score will be the outcome variable.

#### Quality of Life Enjoyment and Satisfaction Questionnaire – Short Form (Q-LES-Q-SF) [66]

The Q-LES-Q-SF is a 16-item self-report questionnaire that assesses quality of life across areas of daily functioning. Total score will be used as the outcome variable.

#### Patient-Reported Outcomes Measurement Information System Global 10 (PROMIS Global 10) [67]

The PROMIS Global-10 consists of 10 items that assess overall perceived quality of life (QOL), including physical health, mental health, social health, pain and fatigue. Total QOL score will be used as the outcome variable.

#### Kessler Psychological Distress Scale Plus (K10+) [68]

The K10+ is a 14-item self-report measure that assesses if a person has been affected by anxiety or depression in the last four weeks. The outcome variable will be total score.

#### Goal Attainment Scaling 2.0 (GAS 2.0) [69]

GAS 2.0 is a revised version of Goal Attainment Scaling (GAS), a technique for quantifying the achievement (or otherwise) of goals set [70]. GAS 2.0 was developed to more objectively determine the level to which a client has attained their goal. Whereas conventional GAS requires the goal setter to estimate the likely outcome (primary goal) in advance, GAS 2.0 derives the primary goal on the basis of: a) current level of functioning and b) maximum realistic level of functioning. It makes use of a goal menu to facilitate and expedite the process of goal setting. The outcome variable will be post-TAU/CR GAS score.

#### Australian Treatment Outcomes Profile (ATOP) [71]

The ATOP is a structured interview that has been validated to measure treatment outcomes in Australian drug and alcohol populations. It contains two sections. Section one details the quantity and frequency of substance use. Section two details health and wellbeing variables such as days in paid work/study, homelessness, eviction risk, violence and arrest, as well as ratings of psychological health, physical health and overall quality of life. This measure will be administered on intake, with participants asked to respond to questions about their substance use, health and wellbeing for the four-week period leading to their commencement of residential rehabilitation. The measure will also be administered at post-discharge follow up, with participants asked to respond to questions about their substance use, health and wellbeing in the previous four weeks.

The following scale will be administered at the end of each session during the CR phase:

#### Group Session Rating Scale (GSRS) [72]

The GSRS is a four-item visual analogue scale, designed to be a brief clinical tool to measure group-therapy alliance. The items are presented as bipolar anchors requiring a response on a ten centimetre line. The scale assesses aspects of individual’s perceptions of a group’s therapeutic environment, including; 1) the relationship between the leader and/or group, 2) goals and topics, 3) acceptability of the approach, and 4) overall fit. GSRS scores are obtained by measuring the marks made by the client and summing the lengths to the nearest centimetre on each of the four lines. Scores are summed out of a total possible score of 40. The total score will be the process variable.

#### Economic analysis

An evaluation of the cost of delivering CR versus TAU will be undertaken.

### Intake measures

The following data will be gathered on intake in the form of a structured questionnaire

1. Age;
2. Highest level of educational attainment;
3. Employment status;
4. Marital status;
5. Number of Children/Dependents
6. Primary substance of misuse;
7. Years of regular use (by substance);
8. Nicotine Use
9. Use of OST
10. Relapses in residential treatment;
11. Relapses in community treatment;
12. Abstinence duration;
13. Country of birth
14. First language
15. Australian/Torres Strait Islander Status
16. Referral source (e.g. self-referred or court-ordered enrolment)

#### Drug and Alcohol Cognitive Impairment Screening Tool (DACIST) [73]

The DACIST was developed to measure self-reported historical factors that may reasonably contribute to cognitive impairment in a SUD population. Items assess history of head injury, overdose, seizures, hospitalization, foetal drug or alcohol exposure, neurological conditions, learning or behavioural difficulties during schooling and subjective appraisal of cognitive impairment.

#### Mini International Neuropsychiatric Interview V7.0.2 for DSM-5 (MINI) [74]

The MINI is a structured interview used for assessing indicators of DSM-5 diagnoses.

### Statistical methods

#### Sample size estimation

The primary outcome is the BRIEF-A GEC difference score between pre- and post-TAU/CR phase. Given previous data for a more comprehensive 24 h CR program [42], sample size calculations were based on a difference in BRIEF-A score of 2.25 for the current 12 h program. We used the sample size methodology for stepped wedge designs put forward by Hooper et al. [75]. Assuming a standard deviation of 5.15, obtained from the Marceau data [42], an individually randomised trial would require a total sample of 165 people in order to have 80% power to detect the intervention effect at the 5% significance level. The design effect for cluster randomisation, assuming an intraclass correlation of 0.05 and anticipating 20 people per cluster, is 1.95 and the design effect for repeated assessment, assuming a cluster autocorrelation of 0.5, is 0.534. The required number of clusters for this stepped wedge design is therefore nine. In order to account for a 10% loss of the total sample to follow-up, (e.g participants that are discharged and not replaced by new participants in the research) and to protect against the possibility of a cluster withdrawing from the trial, an additional cluster will be recruited yielding a total of 10.

### Analysis plan

Baseline characteristics will be summarised using means and standard deviations or frequencies and percentages as appropriate. The primary outcome variable will be the BRIEF-A GEC score and the effect of the intervention will be estimated using a linear mixed model. The model will include the baseline BRIEF-A GEC as a covariate and a fixed effect for each time period after baseline to account for any secular trends. We will also include random effects for treatment centre, time within centre and individual within centre to account for the clustering, repeated measurements on centre and repeated measures on individuals, respectively. The main predictor of interest will be a pre−/post-intervention variable, which will measure the overall effect of the intervention. The data will be analysed according to the intent-to-treat principle, with sensitivity analysis undertaken to include all participants under an appropriate multiple imputation model for the missing data.

Two analyses are proposed to investigate the impact of CR on treatment retention, being:

1. Direct analysis of retention (proportion of time spent in treatment relative to program duration) across TAU and CR conditions; and
2. Comparison of historical retention data (dating back 2–5 years) with retention rates following the first CR phase for each site. A 10% increase in service retention rate will be the indicator of a clinically meaningful gain.

## Discussion

EF impairment is a significant predictor of treatment drop-out for individuals with SUD. There is an opportunity for AOD treatment providers to address cognitive impairment as part of routine care in order to potentially increase treatment efficacy and reduce treatment drop-out. The present study aims to examine the effectiveness of providing a group-based compensatory CR intervention as an adjunct to residential AOD treatment programs. It is expected that compared to pre-intervention, participants will demonstrate a significant improvement in EF and the duration of treatment retention. In addition, it is anticipated that there will be reductions in harmful AOD use and associated health service utilisation, as well as significant improvements in personal goal achievement, quality of life, and treatment satisfaction. As the current study is the first stepped wedge cluster randomised trial of a compensatory CR intervention within a residential AOD population, the results potentially hold important implications for the way that interventions are delivered across treatment settings.

### Strengths and limitations

The current study is a large, multi-site, stepped wedge cluster randomised trial that will be conducted across ten residential treatment programs. The strength of conducting this type of “real world” research is that it is more representative of actual clinical practice and helps to provide some evidence regarding the feasibility of using these types of interventions as part of ongoing routine care. The research design also includes additional attempts to increase the ecological validity of the results by using very inclusive eligibility criteria, including individuals with previous traumatic brain injuries and psychiatric comorbidities.

Another strength of the study is that outcome measures have been carefully chosen to reflect the effects of CR on real-world functioning. Given that performance- and inventory-based EF assessments are minimally correlated and may assess distinct components of EF, the current study aims to provide a comprehensive assessment of EF to account for abilities that are likely to translate to functional outcomes [36, 76]. This study will also be the first to evaluate whether compensatory CR will result in improved treatment retention, which has been shown to be an important predictor of successful long-term outcomes for individuals with SUD [3]. Furthermore, the current study will take person-centred outcomes into account, including measurements of AOD use, health service use, personal goal attainment, quality of life, and treatment satisfaction. Inclusion of person-centred outcomes will measure whether CR is likely to result in contextually meaningful and desired treatment outcomes. Finally, an economic analysis will be undertaken to determine the cost of delivering CR versus TAU in residential treatment settings.

A significant challenge in this trial is the high rate of unplanned drop-out that is common in residential AOD treatment services. According to recent figures, approximately 20% of individuals with SUD unexpectedly cease treatment [2]. To help address this concern, compensatory CR will be delivered in one-hour sessions, twice a week, over a six-week period, which is a reasonable amount of time to expect individuals to dedicate to the intervention. A further challenge will be retaining participants at follow-up. Attempts to improve follow-up rates in the current study include using telephone and online follow-up to administer some outcome assessments, obtaining contact details of significant others to help with locating participants and reinforcing the importance of conducting follow-up to participants.

The current study will be the first stepped wedge cluster randomised trial of a compensatory CR intervention in a residential substance abuse population. The study seeks to address a significant gap in the literature by examining the effectiveness of implementing a six-week CR group program within AOD residential treatment programs. It is expected that following completion of CR, participants will demonstrate significantly improved EF and treatment retention rates, reduced AOD use and health service utilisation rates, as well as achievement of individually-set goals, quality of life, and treatment satisfaction.

## Abbreviations

AOD: Alcohol and other drugs
ATOP: Australian Treatment Outcomes Profile
BEAT: Brief executive assessment tool
BRIEF-A: Behavioural rating inventory of executive function – adult version
BSCS: Brief Self Control Scale
CRiDAS: Cognitive Remediation in Drug and Alcohol Services
DACIST: Drug and Alcohol Cognitive Impairment Screening Tool
EF: Executive functioning
EQ-5D: EUROQOL-EQ-5D-5L
EUROHIS-QOL 8: EUROHIS-QOL 8-item index
GAS 2.0: Goal attainment scaling 2.0
GAS: Goal attainment scaling
GEC: Global executive composite
GMT: Goal Management Training
GSRS: Group Session Rating Scale
K10 +: Kessler Psychological Distress Scale Plus
MINI: Mini international neuropsychiatric interview
OST: Opioid Substitution Therapy
PROMIS Global-10: Patient Reported Outcomes Measurement Information System Global 10
Q-LES-Q-SF: Quality of Life Enjoyment and Satisfaction Questionnaire – Short Form
QOL: Quality of Life
SDMT: Symbol Digit Modalities Test
SDS: Severity of Dependence Scale
SUD: Substance use disorder
TAU: Treatment as usual
